# Analysis of Non-Typeable *Haemophilous influenzae* VapC1 Mutations Reveals Structural Features Required for Toxicity and Flexibility in the Active Site

**DOI:** 10.1371/journal.pone.0112921

**Published:** 2014-11-12

**Authors:** Brooke Hamilton, Alexander Manzella, Karyn Schmidt, Victoria DiMarco, J. Scott Butler

**Affiliations:** 1 Department of Microbiology and Immunology, University of Rochester Medical Center, Rochester, New York, United States of America; 2 Department of Biochemistry and Biophysics, University of Rochester Medical Center, Rochester, New York, United States of America; 3 Center for RNA Biology, University of Rochester Medical Center, Rochester, New York, United States of America; University of Rome Tor Vergata, Italy

## Abstract

Bacteria have evolved mechanisms that allow them to survive in the face of a variety of stresses including nutrient deprivation, antibiotic challenge and engulfment by predator cells. A switch to dormancy represents one strategy that reduces energy utilization and can render cells resistant to compounds that kill growing bacteria. These persister cells pose a problem during treatment of infections with antibiotics, and dormancy mechanisms may contribute to latent infections. Many bacteria encode toxin-antitoxin (TA) gene pairs that play an important role in dormancy and the formation of persisters. VapBC gene pairs comprise the largest of the Type II TA systems in bacteria and they produce a VapC ribonuclease toxin whose activity is inhibited by the VapB antitoxin. Despite the importance of VapBC TA pairs in dormancy and persister formation, little information exists on the structural features of VapC proteins required for their toxic function *in vivo*. Studies reported here identified 17 single mutations that disrupt the function of VapC1 from non-typeable *H. influenzae in vivo*. 3-D modeling suggests that side chains affected by many of these mutations sit near the active site of the toxin protein. Phylogenetic comparisons and secondary mutagenesis indicate that VapC1 toxicity requires an alternative active site motif found in many proteobacteria. Expression of the antitoxin VapB1 counteracts the activity of VapC1 mutants partially defective for toxicity, indicating that the antitoxin binds these mutant proteins *in vivo*. These findings identify critical chemical features required for the biological function of VapC toxins and PIN-domain proteins.

## Introduction

Type II toxin-antitoxin (TA) systems in bacteria first emerged as a mechanism of post-segregational killing caused by plasmid-borne TA operons [1], [2]. Subsequently, the discovery of chromosomally encoded TA systems led to the identification of several apparently distinct mechanisms of action for the encoded toxins [3], [4]. These include ribonucleases that hydrolyze mRNA, rRNA and tRNA, DNA gyrase inhibitors and protein kinases that target translation. In nearly all characterized cases, a single operon encodes the toxin and antitoxin genes, often with closely adjacent or overlapping reading frames allowing translational coupling. The Type II TA operons produce protein toxins and antitoxins, which form complexes that inhibit the activity of the toxin. The TA pair also often acts as a transcriptional repressor of the operon, providing a level of feedback regulation upon which various environmental stimuli may act to derepress TA expression. Upon cellular stress, the antitoxin is cleaved by a protease such as Lon, thereby freeing the toxin from the inhibitor and derepressing expression of the TA operon. The free toxin then carries out its primary function, which results in growth inhibition and eventual cell death [5]. Relief of the stress that led to inactivation of the antitoxin allows its accumulation and inactivation of its cognate toxin, which allows resumption of cell growth. Thus, TA systems have the potential to play a regulatory role in bacterial dormancy, making their study relevant to the problem of pathology caused by bacterial latency. Additionally, stochastic fluctuations in toxin activity within populations of bacteria produce dormant cells resistant to many antibiotics [6], [7]. These persisters can contribute to re-establishment of the bacterial population upon discontinuance of the drug. These characteristics make the elucidation of the mechanisms of TA function an important goal for molecular biologists.

VapC toxins comprise the largest family of Type-II toxins in bacteria [8]. They contain a characteristic PIN-domain found in a wide range of bacteria and eukaryotes where they appear to function as endoribonucleases targeted to the process of protein synthesis or ribosome biogenesis [8]–[10]. Analysis of PIN-domain proteins in bacteria showed that they also exist as single domain proteins (VapC) in TA operons, but their mechanism of action and function remains unclear [9], [11]. One of the two VapC proteins (VapC1) from *H. influenzae* inhibits growth when expressed in *E. coli*, an effect countered by co-expression of its associated antitoxin (VapB1) [12]. Recombinant VapC1 caused RNA degradation *in vitro* and addition of VapB1 inhibited this activity. Likewise, VapC proteins from *Pyrobaculum aerophilum*, and *Mycobacterium tuberculosis* demonstrate sequence specific ribonuclease activity *in vitro* [13], [14]. Recent findings revealed that VapC toxins from *S. flexneri* and *S. enterica* function as initiator tRNA^fMet^ endonucleases, while toxins from *M. tuberculosis* target RNA structures containing a specific sequence motif, the sarcin-ricin loop of 23S rRNA, or inhibit protein synthesis by binding to mRNAs [15].

Along with questions about the targets of VapC toxins, it remains unclear what aspects of their structure, other than four canonical acidic amino acids, contribute to their activity. We addressed this issue using a novel strategy to identify loss of function mutant alleles of the VapC1 toxin from NTHi and discovered numerous amino acid side chains required for its toxicity. Structural modeling places many of these critical groups in proximity to amino acids predicted to participate in the chemistry of the active site of the enzyme. Our findings also support the conclusion that NTHi VapC1, and possibly many other VapC toxins, use an alternative to the canonical active site found in many VapC toxins and PIN-domain proteins. Finally, our findings indicate that mutations that inhibit VapC toxin activity do not necessarily abrogate binding to its VapB antitoxin *in vivo*. These findings identify critical structural features required for the biological function of this important class of bacterial TA systems.

## Materials and Methods

### Bacterial strains and growth conditions

All growth assays were conducted in *E. coli* LMG194 (F- ΔlacX74 gal E thi rpsL ΔphoA (Pvu II) Δara714 leu::Tn10) grown in M9 media [16] supplemented with 50 µg/ml ampicillin and 0.2% glycerol. Growth was monitored in 96-well microtiter plates using a Bio-tek Powerwave XS, which measured A_600_ every 15 minutes at 37°C.

### Molecular cloning

Plasmids expressing VapC1, or VapC1 and VapB1, were constructed by inserting PCR synthesized DNA between the Nco1 and Xba1 sites of pBAD/*Myc*-His B (Invitrogen), pBAD-eGFP-EcMax or pBAD-sfGFP using standard techniques. PCR primer sequences are available upon request. All inserts were subjected to DNA sequence analysis to verify their identity. pBAD-eGFP-EcMax was constructed by insertion of a chemically synthesized, codon optimized eGFP DNA, digested with Xba1 and Spe1, into the Xba1 site of pBAD/*Myc*-His B. pBAD-sfGFP was constructed by insertion of a PCR product derived from the Super Folder GFP plasmid (Sandia Biotech), digested with Xba1 and Spe1, into the Xba1 site of pBAD/*Myc*-His B. pBAD-eGFP-EcMax and pBAD-sfGFP were constructed with 6x-glycine linkers at the N-termini of GFP and a C-terminal fusion to the *Myc*-6× His coding sequence.

### Mutagenesis

VapC1 mutations were created by PCR or oligonucleotide directed site-specific mutagenesis. For PCR mutagenesis, VapC1 was amplified by PCR from pBAD-VapC1 DNA template in five 50 µl reactions containing 20 mM Tris-Cl (pH 8.4), 50 mM KCl, 5 mM Mg_2_Cl, 1 mM dNTP, 25 pmoles DNA primers, 1 µg DNA template and 0.1 unit Taq polymerase. Reactions were run at 94°C, 4 mins.; 30×(94°C, 30 sec., 55°C, 30 sec., 72°C, 1 min.); 72°C, 5 mins. Reactions were pooled, and primers and small molecules removed using a PCR purification kit (Qiagen). The DNA was digested with Nco1 and Xba1 and inserted by ligation into the same sites in pBAD-eGFP-EcMax. The ligation mixtures were transformed into LMG194, plated on Luria Broth agar plates containing 50 µg/ml ampicillin and 0.02% arabinose and incubated for 16 hours at 37°C. Colony GFP fluorescence was analyzed on a Typhoon 9410 imager (GE Biosciences) using the 488 nM blue laser for excitation and the 520BP40 emission filter. Plasmids were recovered from fluorescent colonies and re-transformed into LMG194 to confirm phenotypes. The insert portion of the plasmid DNAs from putative mutants were sequenced (Genewiz) before and after transfer of the VapC1 portion into the Nco1 and Xba1 sites of pBAD-sfGFP to verify that only the desired mutations were present.

Oligonucleotide directed site-specific mutagenesis was carried out by a modification of the method of Fisher and Pei [17]. PCR was carried out in a 50 µL reaction containing 10 µL HF iProof Buffer (Bio-Rad), 1 µL forward mutagenic primer (10 nM), 1 µL reverse mutagenic primer (10 nM), 1 µL 10 mM dNTP's, 4.5 µL DMSO, 0.5 µL HF iProof Polymerase (2 units/µL; Bio-Rad) and 10 ng VapC1 DNA template. Reactions were run at 98°C, 3 mins.; 30×(98°C, 30 sec., 65°C, 30 sec., 72°C, 3 min.); 72°C, 5 mins. Twenty units of DpnI (NEB) was added to the reaction and incubated at 37°C for 30 mins. to destroy DNA template strands. Ten µL of the reaction mixture was transformed into Top10 and colonies selected on LB plates containing 50 µg/ml ampicillin at 37°C. The insert portion of the plasmid DNAs from putative mutants was sequenced (Genewiz) to verify that only the desired mutations were present.

### Western blot analysis

Cells were grown at 37°C in M9 media supplemented with 50 µg/ml ampicillin and 0.2% glycerol to an A_600_ of 0.3. L-arabinose was added to a final concentration of 0.02% and the incubation continued for 2 hours. Cells were collected by centrifugation and the pellets resuspended in 1× SDS PAGE sample buffer and boiled for five minutes before separation of the proteins by SDS-polyacrylamide gel electrophoresis. Proteins were transferred by electrophoresis to nitrocellulose paper and probed with anti-myc antibody (Invitrogen) or antiGroEL antibody (Sigma) prior to detection with secondary antibodies conjugated with horseradish peroxidase and chemiluminescence reagents.

## Results

### NTHi VapC1 functions as a GFP fusion protein *in vivo*

**Figure 1:**
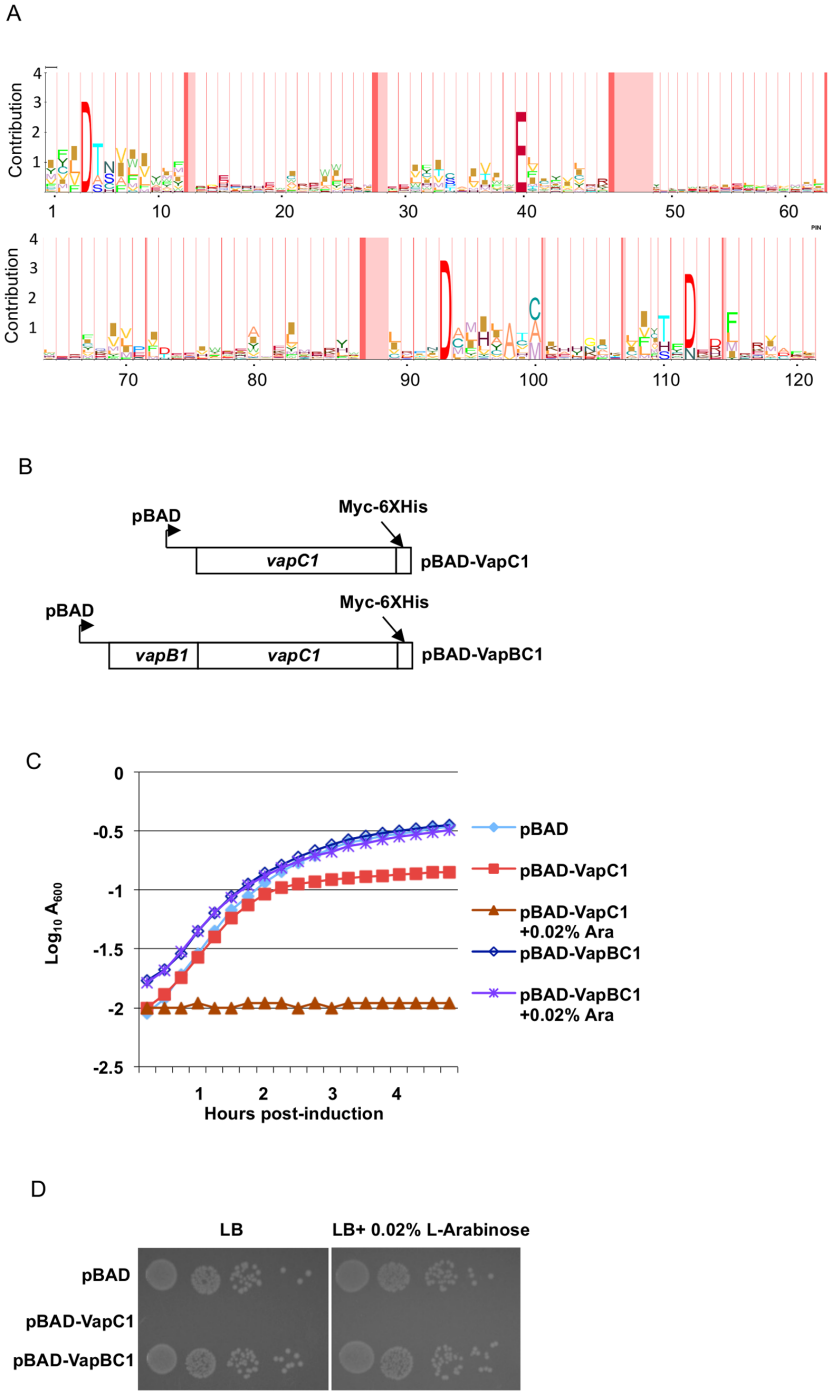
Characteristics of NTHi VapBC1 toxin-antitoxin system *in vivo*. *A*. HMM Logo analysis of 8807 VapC sequences in the PF01850 Pfam family database. The height of letters at each position represents the deviation of that letter's frequency from the background frequency of that letter [18]. *B.* Diagram of *vapC1* or *vapBC1* sequences cloned into pBAD-MycHisB plasmids under control of the L-arabinose inducible pBAD promoter. *C*. Analysis of the growth of LMG194 cells in M9 glycerol with 50 µg/ml ampicillin after induction by addition of L-arabinose to a final concentration of 0.02%. The curves are representative examples of multiple experiments that yielded the same results. *D*. Growth of LMG194 carrying the indicated plasmids after spotting ten-fold dilutions of cells on LB or LB +0.02% arabinose plates that were incubated at 37°C for 16 hours.

VapC toxins, sometimes called PIN-domain proteins, belong to the Pfam database family PF01850, which presently contains 8807 sequences. HMM logo analysis [18] of these proteins reveals significant variation in amino acid sequence, but also several areas of high similarity (Figure 1A). Prominently, the family shares four conserved acidic amino acids that structural analyses indicate co-ordinate a metal ion in the active sites of the enzymes [10], [19]. In several cases, mutations altering each of the four conserved acidic amino acids in the active site of VapC proteins resulted in loss of toxicity *in vivo*, yet the requirement for other amino acids remains unclear [20], [21]. Preliminary to mutagenic analysis of a well-characterized VapC1 toxin from NTHi [22], we constructed plasmid vectors that allow conditional expression of the toxin, or both the toxin and antitoxin from the inducible L-arabinose operon promoter (pBAD) and monitored their effects on cell growth (Figure 1). In each case the plasmids express VapC1 as a *myc*-epitope-6× histidine fusion protein and in pBAD-VapBC1, VapB1 and VapC1 are translated as a single transcript that encodes the two open reading frames in their natural arrangement, which allows translational coupling of VapC1 to VapB1 (Figure 1B). As expected, induction of toxin expression with L-arabinose (pBAD-VapC1 +0.02% Ara) leads to growth inhibition in liquid culture (Figure 1C) and on Petri plates (Figure 1D). Because of leaky expression of VapC1, the strain expressing the toxin from the Ara promoter results in poor growth on LB plates even in the absence of the inducer. Expression of the toxin and the antitoxin (pBAD-VapBC1 +0.02% Ara) does not result in significant growth inhibition, indicating that the antitoxin binds to and inactivates the toxin (Figure 1C). Toxicity of VapC1 does not require the presence of *lon*, indicating independence of these phenotypes from Lon-dependent activation of endogenous TA systems (data not shown).

**Figure 2:**
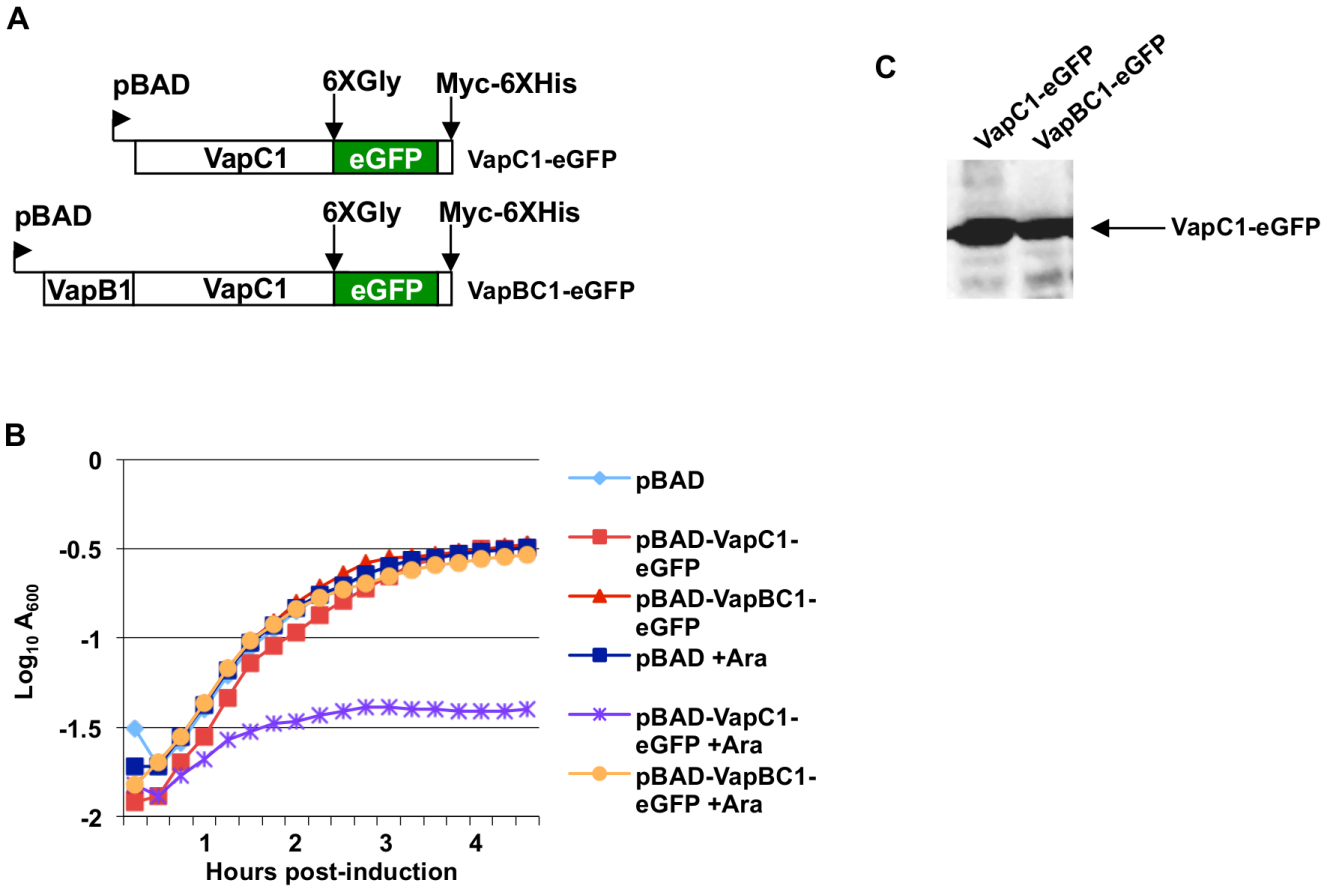
Growth characteristics of VapC1-eGFP fusions. *A*. Diagram of *vapC1* or *vapBC1* sequences cloned as *vapC1*-eGFP fusions into pBAD-MycHisB plasmids under control of the L-arabinose inducible pBAD promoter. *B*. Analysis of the growth of LMG194 cells in M9 glycerol with 50 µg/ml ampicillin after induction by addition of L-arabinose to a final concentration of 0.02%. The curves are representative examples of multiple experiments that yielded the same results. *C*. Western blot analysis of VapC1-eGFP from cells grown as in *B* after a 30-minute induction with L-arabinose to a final concentration of 0.02%. Each lane contains lysate from an equal number of cells.

Next, we constructed a C-terminal fusion of VapC1 toxin to eGFP with an intervening 6X-glycine linker (Figure 2A). We asked if the VapC1-eGFP fusion functions as a toxin *in vivo* and found that expression of the fusion protein inhibits cell growth (Figure 2B). We then asked if co-expression of the antitoxin relieves growth inhibition caused by the VapC1-eGFP fusion, and found that cells grow upon co-expression of the antitoxin (Figure 2B) despite expression of the toxin fusion protein in the cells (Figure 2C). These findings show that; (i) the experimental system reflects the known activities of the toxin and antitoxin on cell growth (Figure 1) [12], (ii) the VapC1-eGFP fusion retains its toxin activity and (iii) VapB1 acts effectively as an antitoxin for the eGFP fusion protein.

### Identification of VapC loss of function mutations

**Figure 3:**
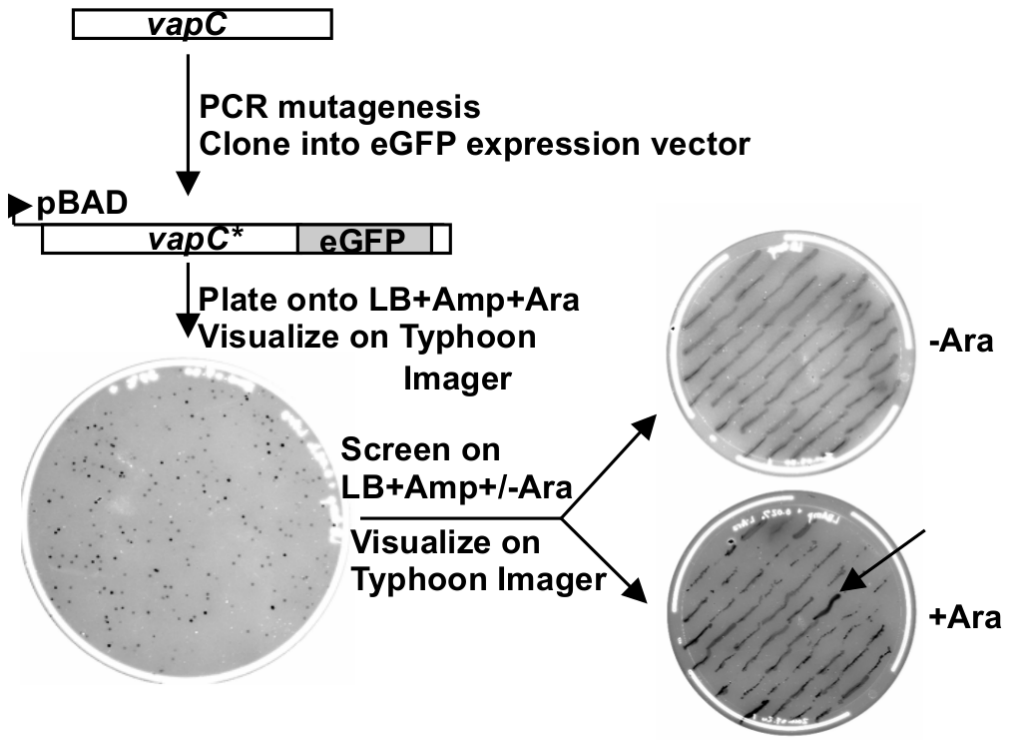
Workflow for isolation of VapC1 loss of function mutations. VapC1 DNA was amplified by PCR with Taq polymerase and inserted in frame with eGFP in pBAD-eGFP-MycHisB using standard DNA cloning techniques. DNA was transformed into Top10 and plated on Luria Broth + ampicillin (50 µg/ml; LBA) plates containing 0.02% L-arabinose. After incubation of the plates at 37°C for 16 hours, colonies were visualized on a Typhoon 9410 imager (excitation at 488 nm; 520BP40 emission filter). Colonies were then screened on LBA plates with and without arabinose, and colonies that grew on both plates and exhibited fluorescence above background were chosen for further analysis.

To identify amino acids required for VapC toxicity we employed; (i) PCR mutagenesis, (ii) selection for loss of growth inhibition upon induction of expression and (iii) screening for GFP fluorescence to identify mutations that inactivate VapC1-eGFP (Figure 3). Initial attempts at selecting such mutants with VapC lacking the fusion to GFP yielded several mutations (T7P (twice) and E120G), but mostly nonsense mutations. Use of VapC1-eGFP and the screen for GFP fluorescence allowed us to avoid nonsense mutations in VapC1, since these produce truncated VapC1-eGFP polypeptides with background levels of fluorescence (Figure 3). Strains that produce full length, defective VapC1-eGFP grow in the presence of inducer and exhibit substantial fluorescence (arrow, Figure 3). This method yielded 23 isolates with single mutations causing defects in VapC1-eGFP (Table 1). This includes T7P (twice) and E120G, as well as 7 double mutants, two of which contain changes to E120 and one each changing F121, N117 and E43 (Table 1). The fact that the selection yielded the T7P and E120G mutations independently in the *vapC1* and *vapC1-eGFP* selections supports the conclusion that the activity of the GFP fusion protein reflects that of the VapC1.

**Table 1:** *VapC1* mutations isolated in this study.

Isolated as *vapC1-GFP*		Isolated as *vapC1*	
Mutant	Genotype	Mutant	Genotype
M1-2-A	S37G	10-M2-9	T7P
M1-2-C	T7P	17-M3-7	L88R
M1-2-D	E120G	9-M2-8	T7P
M1-2-E	E120K, I61V	M4-4	E120G
M1-3-A	A103V		
M1-3-B	A103V		
M1-4-B	N9H, A23V		
M1-4-C	A85P		
M1-4-D	H105R, K19E		
M1-4-E	A103T		
M1-4-I	F121S		
M1-5-B	N110I, I124T		
M1-5-C	S37G		
M1-6-B	E43G, N117Y		
M1-6-C	F121S, I124G		
M1-6-E	S37N		
M1-7-B	D99G		
M1-7-C	T115I		
M1-7-D	C104R		
M1-7-E	R93G, E120G		
M1-7-I	T7P		
M1-7-J	V70A		
M1-8-B	W101C		
M1-8-C	W84R		
M1-8-E	V70A		
M1-8-F	G92V		
M1-8-G	L127H		
M1-8-J	T115A		
M1-1	D6A		
M2-1	E43A		

### VapC1 mutations cause a range of toxicity defects

**Figure 4:**
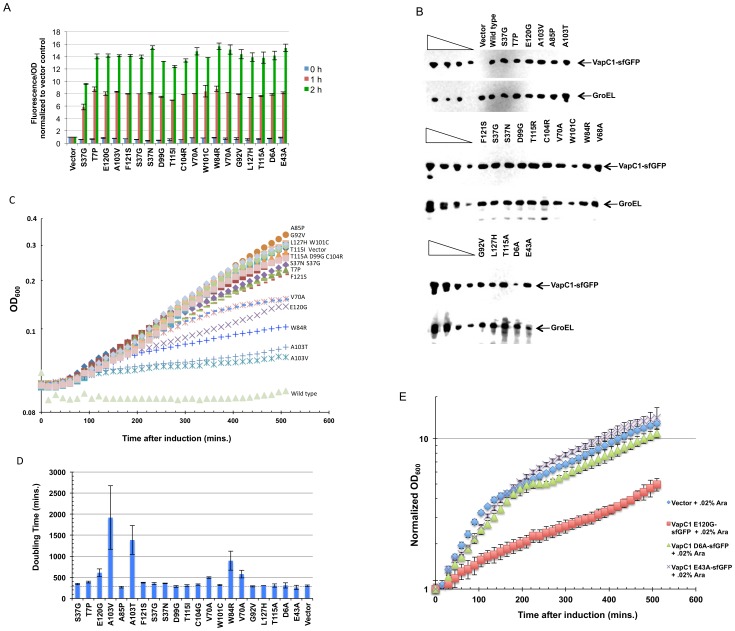
VapC1 mutations cause a spectrum of toxicity defects. *A.* Fluorescence yield from LMG194 cells carrying VapC1 mutants grown in M9 glycerol with 50 µg/ml ampicillin as a function of time after induction with arabinose at a final concentration of 0.02%. Fluorescence was measure in triplicate in a Typhoon 9410 Imager and normalized to the OD_600_ of the culture. *B.* Western blot analysis of VapC1 mutant proteins isolated 60 minutes after induction with arabinose at a final concentration of 0.02%. Blots were probed with anti-myc antibody for VapC1 proteins and anti-GroEL as a loading control. The first four lanes in each panel show a two-fold dilution series of VapC1-sfGFP as a control for signal linearity. *C*. Analysis of the growth of LMG194 cells expressing the indicated VapC1 mutants in M9 glycerol with 50 µg/ml ampicillin after induction by addition of L-arabinose to a final concentration of 0.02%. The curves are one set of examples of two biological replicates whose average doubling time and standard error is shown in (D). *D.* Doubling times for cells expressing VapC1 mutants. Values represent the doubling times calculated for the first 510 minutes of growth and are the average of two independent biological replicates, shown with error bars representing the standard error. *E*. Analysis of the growth of LMG194 cells expressing the indicated VapC1 mutants in M9 glycerol with 50 µg/ml ampicillin after induction by addition of L-arabinose to a final concentration of 0.02%. Time points on the curves are the average of three biological replicates with error bars representing the standard deviation.

Preliminary western blot analysis indicated that cells express each of the mutant proteins at levels similar to wild-type VapC1. However, each of the mutants displayed weak fluorescence compared to the wild type protein co-expressed with VapB1 antitoxin (data not shown). Previous studies showed that mutations in the non-GFP portion of GFP fusion proteins often interfere with the folding of GFP and decrease its fluorescence. The use of superfolder GFP (sfGFP) often solves this problem as it folds more quickly and independently of its fusion partner [23]. Indeed, replacement of GFP with sfGFP in each of the mutants increased their fluorescence about 10-fold. Based on sfGFP fluorescence and western blot analysis, each of the mutant proteins, with the exception of D6A, shows a similar pattern and extent of induction after addition of arabinose (Figure 4A&B). However, varying degrees of toxicity become apparent upon monitoring cell growth, which indicates that the selection/screening scheme identified mutations with a broad range of VapC defects (Figure 4C&D). We also compared how two of the mutations affect the relative toxicity in the context of VapC1 and VapC1-sfGFP to determine whether the sfGFP fusion might attenuate the toxicity of the VapC1 alleles. The results showed that the fusion enhances toxicity of the E120G allele, but has no effect on T7P (data not shown). This finding supports previous observations indicating that sfGFP enhances the activity of its fusion partners by increasing their solubility [23].

**Figure 5:**
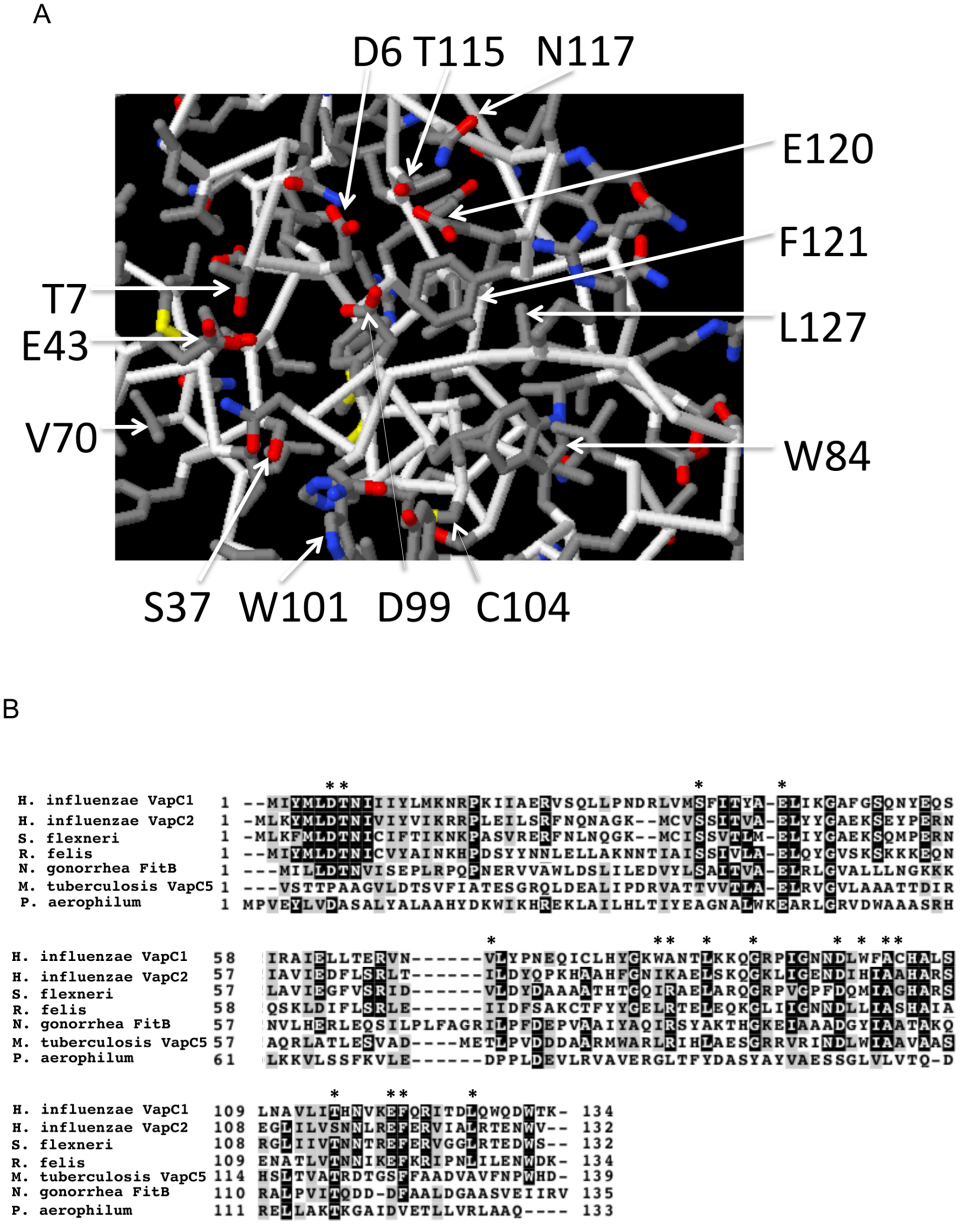
Structural analysis of VapC1 mutations. *A*. Position of mutations affecting activity of NTHi VapC1. NTHi VapC1 was modeled to *Shigella flexneri* VapBC (MMDB ID: 94821; PDB ID: 3TND) using Phyre [25]. Modeling confidence was 100% for 98% of the VapC1 sequence. Arrows indicate mutated NTHi VapC1 side chains. Colors correspond to; white (peptide backbone), grey (amino acid side chains), blue (nitrogen), yellow (sulfur) and red (oxygen). *B*. Comparison of NTHi VapC proteins with several VapC proteins from the listed species. Asterisks indicate the position of NTHi VapC1 mutations described herein. Black boxes indicate identity in at least four of seven species and gray boxes indicate similarity in at least four of seven species.

Thirteen of the VapC1 mutations result in growth rates similar to the empty plasmid vector upon induction suggesting that the changes cause major defects in toxin activity (Figures 4C&D). The normal side chains of these amino acids lie near the active site of the protein based on modeling VapC1 to the crystal structure of the closely related *S. flexneri* VapC [24] (Figure 5A). Comparison of NTHi VapC1 with other VapC sequences predicts that three acidic amino acids (D6, D99, E43) should function in the chemical mechanism of toxin activity by coordinating a metal ion in the active site (Figure 1A and 5B). Indeed, modeling of NTHi VapC1 to *S. flexneri* VapC predicts that all three of these side chains sit in close proximity in the structure [25] (Figure 5A). Nevertheless, the selection/screen only identified D99 as essential for activity. So, we created D6A and E43A by site directed mutagenesis and found that although cells express the mutant proteins, they fail to inhibit growth (Figure 4E). Western blot and fluorescence analyses of these mutants indicate comparable levels of E43A, but low expression of D6A relative to the other mutants, suggesting that lack of activity for the D6A mutant may result from instability of protein. Nevertheless, the findings support previous studies indicating that E43 and D99 play a critical role in the activity of VapC and PIN-domain proteins [20], [21], [26]–[32]. Importantly, the identification of functional defects caused by mutation of conserved side chains, such as T7, S37, T115, F121 and L127 that are predicted to lie near the active site reveals their requirement for the activity of the toxin.

### NTHi VapC toxins utilize a variation of the canonical PIN-domain active site

**Figure 6:**
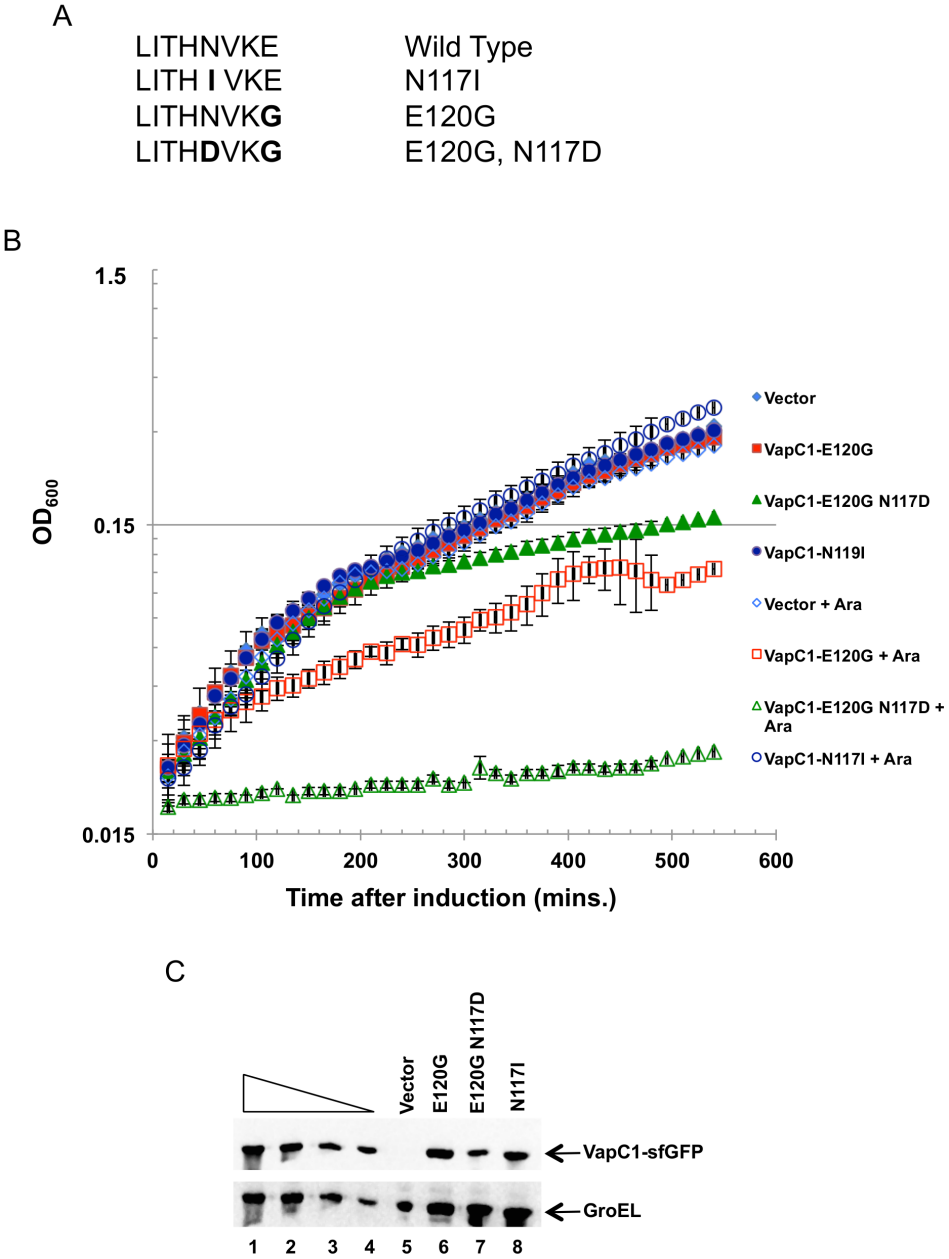
N117D suppresses the E120G defect. *A.* Comparison of the amino acid sequence of VapC1 and mutants from positions 113 to 120. Mutations are indicated in bold. *B*. Analysis of the growth of LMG194 cells expressing the indicated alleles of VapC1 in M9 glycerol with 50 µg/ml ampicillin after induction by addition of L-arabinose to a final concentration of 0.02%. Time points on the curves are the average of three biological replicates with error bars representing the standard deviation. *C*. Western blot analysis of VapC1 mutant proteins isolated 60 minutes after induction with arabinose at a final concentration of 0.02%. Blots were probed with anti-myc antibody for VapC1 proteins and anti-GroEL as a loading control. The first four lanes in the panel show a two-fold dilution series of VapC1-sfGFP as a control for signal linearity.

In VapC proteins, the fourth of four conserved acidic residues is almost always aspartate, which lies in a conserved motif; L/V, X, S/T, X, **D** [33]. However, the sequence in NTHi VapC1 and VapC2, and many other proteobacteria is L, X, S/T, X, **N** (Figure 5B&6A). The polar asparagine cannot participate directly in the inferred coordination of a metal cation required for the chemistry of the active site, and mutation of aspartate at this position in other PIN-domain/VapC proteins inactivates the enzymes [27], [28], [32]. However, the VapC1 glutamate at position 120 is conserved in bacterial species closely related to NTHi that also lack the canonical aspartate (Figure 5B). Moreover, modeling suggests that the E120 side chain projects into the putative active site in close proximity to D6, E43, and D99 (Figure 5A). Thus, the use of E120 instead of D at position 117 may define an uncharacterized variant active site for VapC-proteins (Figure 5B&6A). In support of this hypothesis, the E120G mutation significantly reduces toxicity of VapC1 (Figures 4C, D&6B). The conservation of N117 in proteobacteria implies its requirement for the activity of the toxin. We tested this by mutating the polar asparagine to a non-polar isoleucine. This mutation inactivates VapC1, consistent with a requirement for a polar side chain at this position (Figure 6A&B). Additionally, the predicted proximity of N117 to the basic amino acids in the active site raised the possibility that its conversion to aspartate might allow it to function in place of E120, as apparently it does in other VapC proteins. Accordingly, we asked whether conversion of N117 to aspartate would restore the activity of the defective E120G toxin. Indeed, the double mutant, VapC1- E120G N117D created by site-directed mutagenesis grows poorly compared to VapC1-E120G, suggesting that it functions effectively as a VapC toxin (Figure 6A&B). In each case, western blot analysis reveals that cells express the mutant proteins upon induction (Figure 6C).

### VapC1 mutations that alter toxicity do not abolish interaction with VapB1

**Figure 7:**
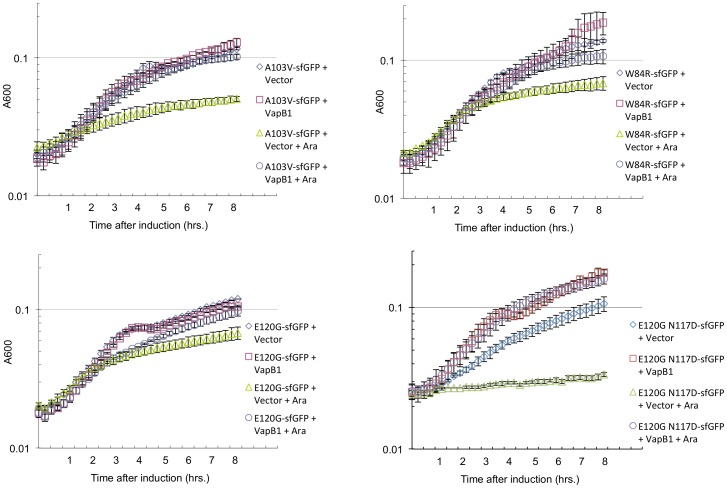
Ability of antitoxin VapB1 to relieve toxicity of VapC1 mutants. *A*. Analysis of the growth of LMG194 cells expressing the indicated alleles of VapC1 in M9 glycerol with 50 µg/ml ampicillin after induction by addition of L-arabinose and/or IPTG to a final concentration of 0.02% and 0.5 mM, respectively. Time points on the curves are the average of two biological replicates with error bars representing the standard error.

Many of the mutations described here abolish the toxin activity of VapC1 *in vitro*. Consistently, many of the mutations we identified occur at highly conserved residues mapping to the modeled active site region of the enzyme (Figure 5), and all but one of the mutant proteins are stably expressed in the cell (Figure 6C). Nevertheless, we cannot eliminate the possibility that the mutations cause some gross protein folding defect. However, antitoxins generally show strong specificity in binding to their cognate toxins [13], [34], [35]. Analysis of crystal structures of VapB-VapC complexes suggests that the C-terminal third of VapB antitoxins interact directly with VapC toxins [24], [36]–[39]. Interestingly, the most distal portion of VapB binds in the groove of VapC containing the active site of the toxin and VapB side chains appear to interact with VapC amino acids involved in active site chemistry. These considerations strongly suggest that binding of VapB1 to VapC1 requires that the toxin exist in the normally folded state. Accordingly, we tested whether VapB1 could neutralize the toxin activity of several VapC1 mutants *in vivo* by assaying the second activity of the toxins, the ability to specifically bind their antitoxin VapB1. The results show that expression of VapB1 counteracts the toxicity of the A103V, W84R, E120G and E120G N117D VapC1 mutants (Figure 7). These findings reveal that these mutations do not have a major effect on the antitoxin binding activity of VapC1 and support our conclusion that the mutations do not have a major negative effect on the structure of the toxin *in vivo*.

## Discussion

VapC proteins comprise the largest family of toxins in the Type II TA systems of bacteria. Recent studies revealed the function of three members of this family as endonucleases that cleave initiator tRNA^fMet^ in *S. flexneri* and *S. typhimurium*, or the sarcin-ricin loop of 23S rRNA in *M. tuberculosis* [15], [21]. Although the activities of this important class of proteins and their eukaryotic relatives depends critically on four conserved acidic side chains, little functional evidence exists on the importance of other amino acids in prokaryotes, nor the related PIN-domains in eukaryotes. The findings presented here identify critical structural requirements for the biological function of VapC toxins and provide evidence for a conserved, alternative configuration of the active site in the VapC toxins of many bacteria.

Many of the VapC1 mutations identified here eliminate toxicity *in vivo* thereby identifying novel structural requirements for the activity of VapC and PIN-domain proteins [24], [36]–[39]. Arcus et al. suggested that the conserved T7 stabilizes the conformation of metal cation binding side chain D6 by H-bonding interactions. Our results indicate that VapC activity requires T7, consistent with their proposal, as well as the side chain's conservation in most VapC toxins and the PIN-domains of eukaryotic nucleases such as SMG6 and EST1 [28], [40]. We also find a requirement for T115, which is conserved as T or S, typically two residues from the fourth conserved aspartate in VapC toxins (Figure 1A). This amino acid is also conserved in eukaryotic PIN-domain proteins and the structure of SMG6 presented by Glavan et al. indicates that it participates in a H-bond network with acidic amino acids that co-ordinate the essential metal cation in the active site [28]. Thus, T115 likely plays an essential role in stabilizing the configuration of these side chains in the active site, or it may contribute to activation of water for phosphodiester bond hydrolysis [8], [28], [41].

NTHi VapC1 toxicity and the activity of eukaryotic relatives also require some combination of counterparts of D6, E43 and D99, the acidic side chains predicted to co-ordinate the active site metal based on structures of VapC and PIN-domain proteins [21], [26]–[28] (Figure 1A). Our finding of a requirement for D99 is consistent with the results of Cline et al. who found that VapC1 with a D99N mutation no longer functioned as a toxin, but could interact with VapB1 to form a complex that binds the VapBC1 promoter [42]. Interestingly, our findings indicate that VapC1 from NTHi employs E120 rather than the aspartate conserved in many other VapC toxins at position 117, the fourth canonical acidic side chain thought to play a role in stabilizing the catalytic metal ion via a water bridge [33]. E120 also exists in NTHi VapC2 as well as the VapC toxins from *R. felis* and *S. flexneri*, and VapC toxins from many species of proteobacteria lacking the counterpart of D117 (Figure 5B). As in the modeling to the *S flexnerii* VapC, E120 of NTHi VapC1 projects into the active site of the structure, modeled by Phyre2 to the *R. felis* VapC, consistent with evidence suggesting that it makes hydrogen bond contacts with the conserved equivalents of N8 and T115 in *R. felis* VapC [36]. These observations support the conclusion that this alternative acidic side chain plays an essential role in the function of VapC toxins in a wide variety of bacteria. Indeed, we found that mutation of asparagine at 117 to aspartate largely compensates for loss of E120 in VapC1, suggesting a certain degree of flexibility in the active site of these toxins. The fact that expression of VapB1 inhibits the toxicity of VapC1 E120G and E120G, N117D suggests that these mutations do not cause major alterations in the structure of the toxin. Interestingly, *M. tuberculosis* VapB20 has the canonical aspartate in a position similar to canonical VapC proteins, while *S. flexneri* and *S. typhimurium* have the alternative equivalent of E120. It remains unknown whether this difference governs the substrate specificity of VapC20 for 23S rRNA and the VapC toxins from *S. flexneri* and *S. typhimurium* for initiator tRNA^fMet^.

Our analysis also revealed a dependence of VapC1 on several other side chains not previously predicted to play an essential role in the activity of VapC toxins. These include the aromatic amino acids W84, W101 and F121. Structural modeling predicts that these side chains lie near the active site suggesting that they could play a role in RNA substrate binding via stacking interactions with nucleobases (Figure 5A). Indeed, the model positions the conserved F121 facing into the active site consistent with a role in positioning RNA for hydrolysis.

Crystal structures of VapB-VapC complexes indicate that the C-terminal third of VapB antitoxins interact with a cleft in the VapC toxins that includes the active site [24], [36]-[39]. Analysis of the structures suggests that interaction between VapC and VapB partners requires side chains necessary for VapC toxin activity. Despite the critical nature of these contacts for the biological fuction of VapBC pairs, no functional evidence exists to support this model. We tested this in the cases where VapC1 mutations diminished, but did not eliminate toxicity *in vivo*. The results indicate that none of these mutations has a measurable effect on the ability of the VapB1 to block the toxicity of the VapC mutants *in vivo*. In the case of, A103V, W84R, E120G, and the double mutant E120G N117D, expression of VapB1 fully counteracts VapC1 toxicity. The ability of VapB1 to interact functionally with these mutant toxins suggests that, despite their important roles in VapC1 toxicity, these amino acids do not play an essential role in the interaction with the antitoxin. Moreover, the fact that VapB antitoxins make specific contacts in and around the active site of their cognate toxins supports our conclusion that the ability of VapB1 to recognize and suppress the toxicity of mutant VapC1 *in vivo* indicates that these mutations do not cause significant misfolding of the toxin.

In summary, the findings reported here reveal critical structural features required for the biological function VapC toxins, and PIN-domain proteins in general. The results support the conclusion that the activity NTHi VapC1 requires a novel, alternative active site motif that is likely employed by many other members of this toxin family. This raises the intriguing possibility that VapC target specificity may be governed by small differences in the enzymes' active sites.
